# Successful conversion surgery after FOLFIRINOX therapy in a patient with advanced pancreatic acinar cell carcinoma with a solitary peritoneal dissemination: A case report

**DOI:** 10.1002/cnr2.1648

**Published:** 2022-06-06

**Authors:** Sunao Uemura, Hiromichi Maeda, Nobuhisa Tanioka, Sachi Yamaguchi, Masaya Munekage, Hiroyuki Kitagawa, Tsutomu Namikawa, Shota Yamamoto, Takuhiro Kohsaki, Mitsuko Iguchi, Kazushige Uchida, Kazuhiro Hanazaki

**Affiliations:** ^1^ Department of Surgery Kochi Medical School Nankoku Japan; ^2^ Department of Gastroenterology and Hepatology Kochi Medical School Nankoku Japan; ^3^ Department of Endoscopy Kochi Medical School Nankoku Japan; ^4^ Department of Pathology Kochi Medical School Nankoku Japan

**Keywords:** conversion surgery, FOLFIRINOX, pancreatic acinar cell carcinoma, solitary peritoneal dissemination

## Abstract

**Background:**

Pancreatic acinar cell carcinoma is rare; it accounts for 1% of all malignant pancreatic exocrine tumors. Although surgical resection is an option for curative treatment, the safety and efficacy of conversion surgery in patients with pancreatic acinar cell carcinoma with metastasis remain unknown.

**Case:**

A 67‐year‐old man with epigastric pain and a pancreatic tumor was referred to our hospital. Computed tomography revealed a large tumor with a maximum diameter of 67 mm at the pancreatic head and a 23‐mm mass in the left upper abdominal cavity. Because a definitive diagnosis could not be made based on endoscopic ultrasonography‐guided fine needle aspiration biopsy findings, a diagnostic laparoscopy was performed. The tumor in the greater omentum at the left upper abdomen, resected under laparoscopy, was histopathologically diagnosed as pancreatic acinar cell carcinoma. Therefore, the pancreatic tumor was diagnosed as an unresectable pancreatic acinar cell carcinoma with a solitary peritoneal dissemination. The size of the main pancreatic tumor decreased to 15 mm after 18 courses of FOLFIRINOX (5‐fluorouracil, leucovorin, irinotecan, and oxaliplatin). Subsequently, the patient underwent conversion surgery, and the initial diagnosis of pancreatic acinar cell carcinoma was confirmed on pathological examination. The patient was discharged 31 days postoperatively, following which he received adjuvant chemotherapy with S‐1. No sign of recurrence has been observed for 32 months after surgical resection.

**Conclusion:**

FOLFIRINOX may be effective in patients with pancreatic acinar cell carcinoma, and conversion surgery after FOLFIRINOX may be applicable to selective patients.

## 1 INTRODUCTION

Pancreatic acinar cell carcinoma (PACC) is a rare pancreatic tumor that comprises tumor cells resembling acinar cells that produce pancreatic enzymes.^1^ The 5‐year survival rate after curative surgical resection of PACC is 44%–72%,^1^, ^2^ which is better than that of pancreatic ductal adenocarcinoma (PDAC). However, 58% of patients with PACC have metastases at diagnosis,^1^ and the 5‐year survival rate decreases to 0%–22% in those with unresectable PACC.^1^, ^2^ Although unresectable PACC shows a better response to chemotherapy than PDAC,^3^ the optimal chemotherapy regimen for treating this tumor has not been established, partly due to its rarity. Therefore, chemotherapy regimens for treating PDAC are often used to treat patients with PACC. These regimens include S‐1 and gemcitabine,^4^ oxaliplatin‐containing regimens,^5^ and FOLFIRINOX (5‐fluorouracil [5‐FU], leucovorin, irinotecan, and oxaliplatin).^6^, ^7^

Providing effective chemotherapy to patients with unresectable cancer may increase the success rate of conversion surgery in them. Herein, we present a case of successful conversion surgery for advanced PACC with a solitary peritoneal dissemination after FOLFIRINOX treatment.

## 2 CASE

**FIGURE 1:**
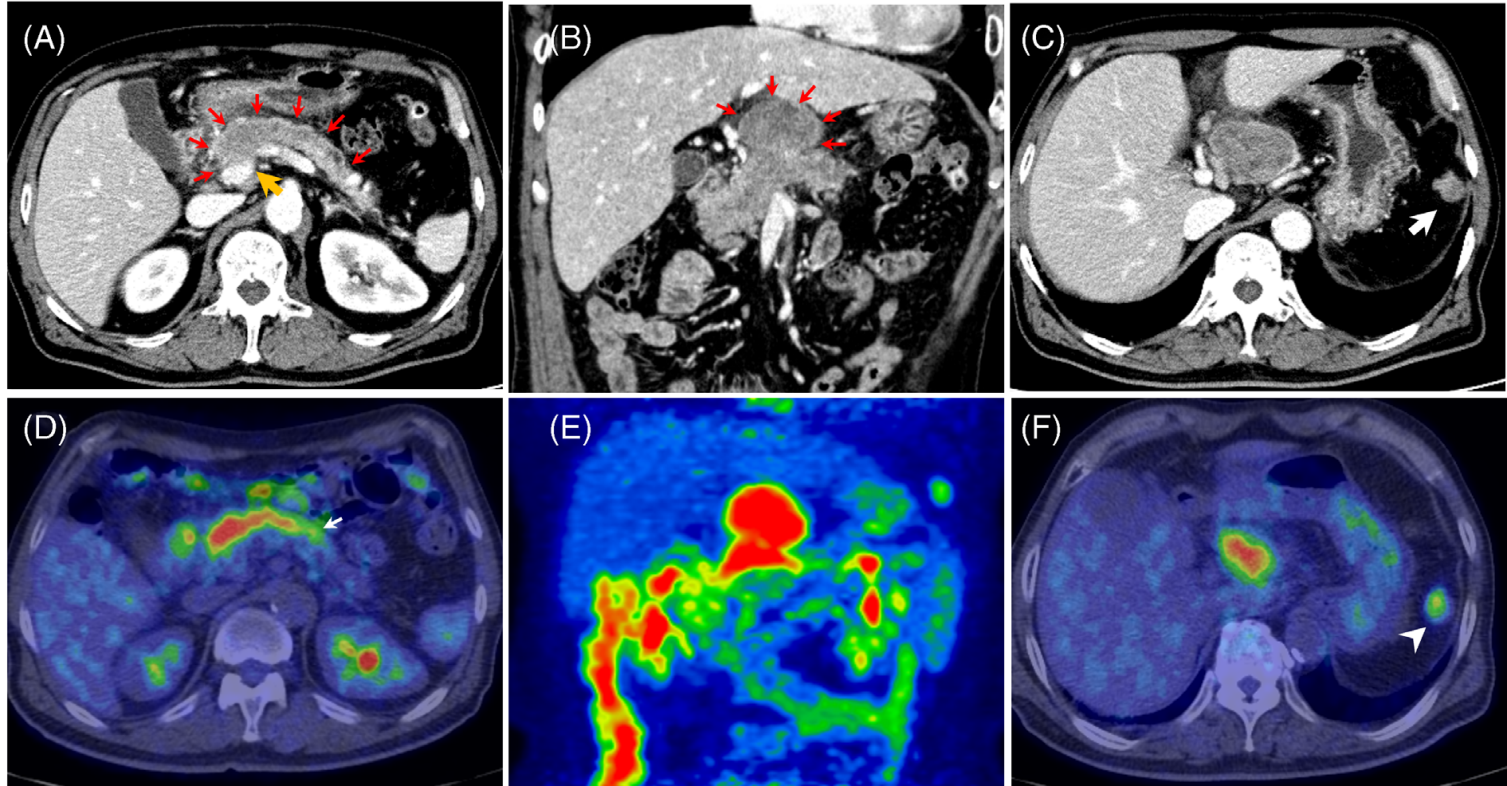
Pre‐treatment images. (A) Abdominal enhanced computed tomography (CT) image showing a pancreatic tumor with growth to the pancreatic body and tail (*red arrows*). The tumor shows slight contact (less than 180° without deformity) with the portal vein (*orange arrow*). (B) Coronal‐view image showing a large tumor with a maximum diameter of 67 mm and remarkable extrapancreatic invasion (*red arrows*). (C) A 23‐mm mass in the left upper abdominal cavity (*arrow*). (D) [^18^F] fluorodeoxyglucose positron emission tomography (FDG‐PET)‐CT showing FDG uptake in the pancreatic tumor (maximum standardized uptake value [SUVmax]: 7.6) with mild FDG uptake in the pancreatic body and tail (*arrow*). (E) Coronal‐view FDG‐PET‐CT image. (F) FDG uptake in the tumor in the abdominal cavity (SUVmax: 3.4; *arrowhead*)

**FIGURE 2:**
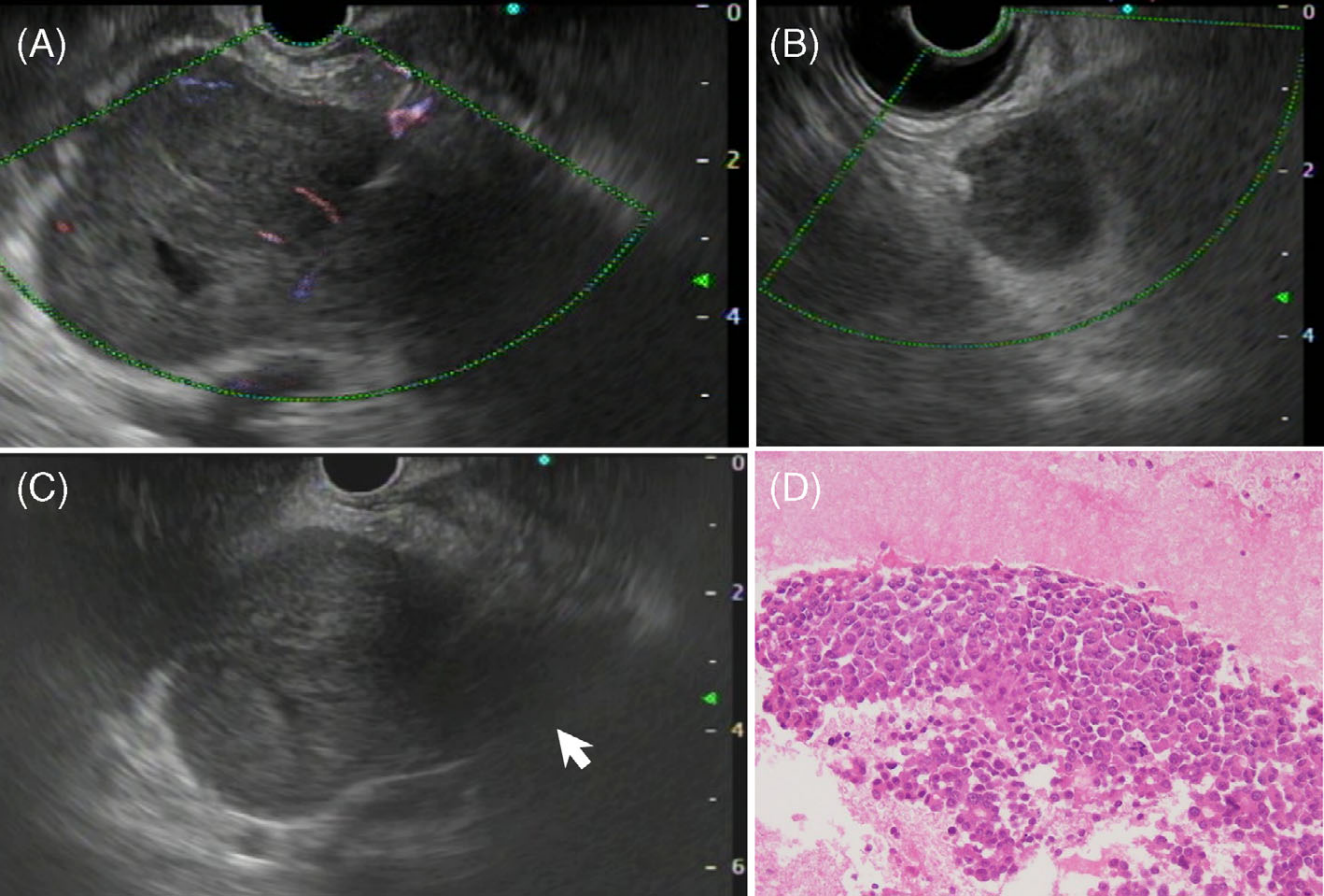
Endoscopic ultrasonography and pathological specimen. (A) Endoscopic ultrasonography (EUS) showing a hypoechoic mass with a smooth surface and weak flow signals in the pancreatic and (B) the intra‐abdominal tumors. (C) The pancreatic tumor shows infiltration to the pancreatic body and tail in the main pancreatic duct (*arrow*). (D) Hematoxylin and eosin staining of the pathological specimen obtained through EUS‐guided fine needle aspiration reveals dense proliferation of tumor cells with a high nuclear‐cytoplasmic ratio, eosinophilic granular vesicles, and round nuclei with slightly poor binding

A 67‐year‐old man presented to a local hospital with a complaint of epigastric pain for 2 days and body weight loss of 5 kg for 6 months. Laboratory test results showed elevated levels of C‐reactive protein (7.23 mg/dl), white blood cell count (9400/μl), and urinary amylase (739 U/L; normal range: 50–500 U/L), leading to a diagnosis of acute pancreatitis. After treatment with antibiotics and gabexate mesilate for 5 days, he was referred to Kochi Medical School Hospital due to having a pancreatic tumor on computed tomography (CT). Laboratory test results in our hospital showed normal levels of serum amylase and tumor markers such as carcinoembryonic antigen, carbonic anhydrase 19–9 (CA19‐9), s‐pancreas‐1 antigen, and duke pancreatic monoclonal antigen type 2, but the serum lipase level was elevated to 1507 U/L (normal range: 13–55 U/L). Contrast‐enhanced abdominal CT revealed a large tumor with a maximum diameter of 67 mm at the pancreatic head with growth to the pancreatic body and tail (Figure 1A) and remarkable extrapancreatic invasion (Figure 1B). The tumor was observed to have slight contact (less than 180° without deformity) with the portal vein (PV) and superior mesenteric vein (SMV), but no contact with the common hepatic artery (CHA) and superior mesenteric artery. Additionally, a 23‐mm mass was identified in the left upper abdominal cavity (Figure 1C). [^18^F] fluorodeoxyglucose (FDG) positron emission tomography (PET)‐CT revealed FDG uptake in the pancreatic tumor (maximum standardized uptake value [SUVmax]: 7.6), with mild FDG uptake noted in the pancreatic body and tail (Figure 1D, E) and in the tumor in the abdominal cavity (SUVmax: 3.4) (Figure 1F). Both tumors were hypoechoic with smooth surfaces and weak flow signals on endoscopic ultrasonography (EUS; Figure 2A, B), and the main pancreatic tumor had infiltrated to the pancreatic body and tail in the main pancreatic duct (MPD; Figure 2C).

**FIGURE 3:**
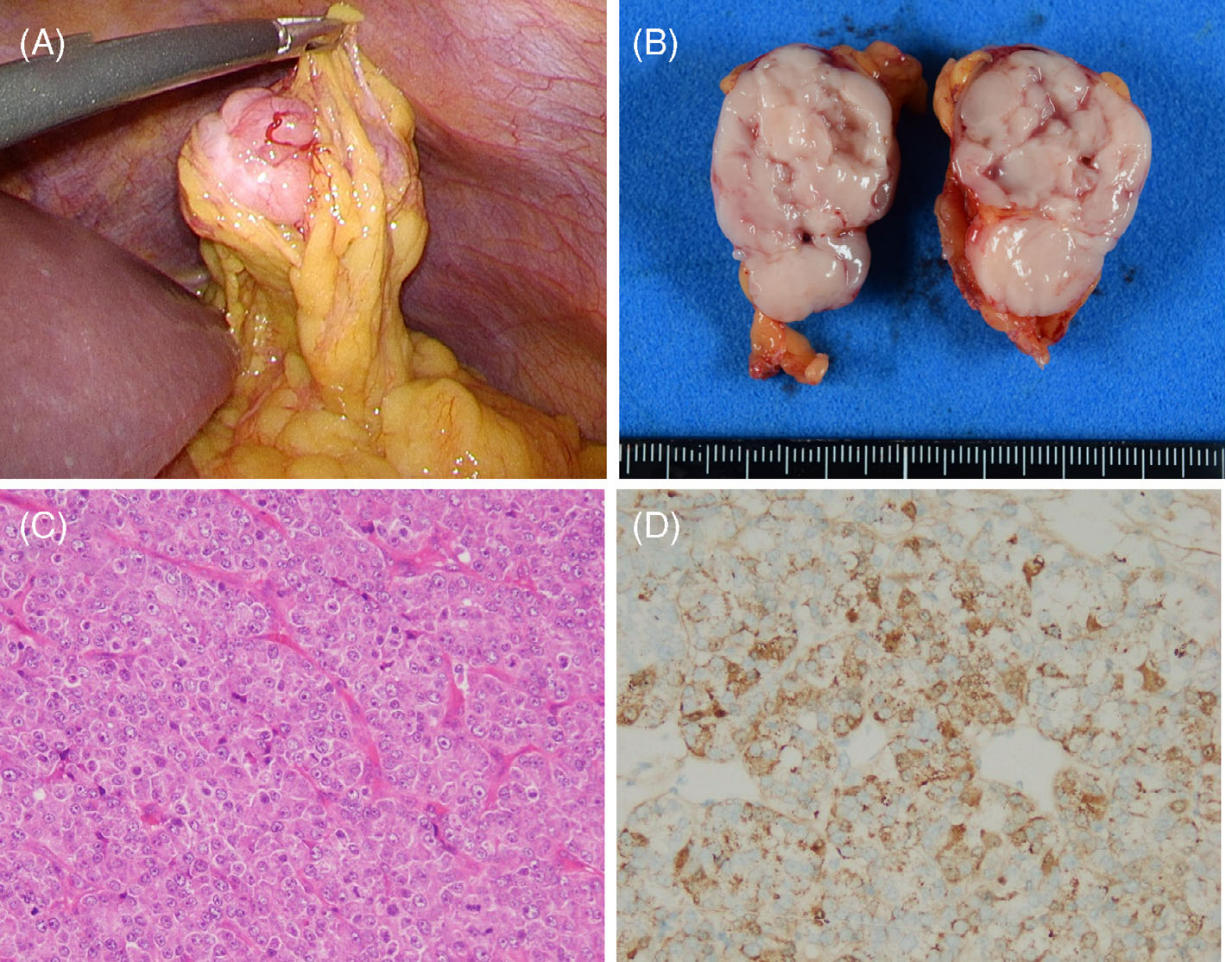
Diagnostic‐staging laparoscopy findings. (A) Surgical findings. The tumor is seen in the greater omentum at the left upper abdomen. (B) Macroscopically, the dissected surface of the resected tumor is solid and pinkish. (C) Hematoxylin and eosin staining reveals atypical cells with swollen nuclei and eosinophilic granular cytoplasm with scant vascular interstitium and sheet‐like proliferation in a scattered rosette‐like arrangement. Multiple mitoses can be observed (12 counts/high‐power field), although coagulative necrosis is not clearly seen. (d) The tumor is positive for trypsin

EUS‐guided fine needle aspiration biopsy revealed that the pancreatic tumor was composed of small round cells with rosette‐like structures (Figure 2D). The tumor was negative for vimentin, chromogranin A, synaptophysin, CD10, CD56, and β‐catenin. Because these findings made definitive diagnosis difficult, diagnostic‐staging laparoscopy was performed. During laparoscopy, there was no liver mass and no ascites, and a peritoneal lavage cytology revealed no cancer cells. The tumor in the greater omentum at the left upper abdomen was resected (Figure 3A, B). Histopathological examination revealed atypical cells with swollen nuclei and eosinophilic granular cytoplasm with scant vascular interstitium and sheet‐like proliferation in a scattered rosette‐like arrangement (Figure 3C). The immunohistochemical findings are shown in Table 1. The tumor was positive for trypsin (Figure 3D), which led to a diagnosis of PACC.

**TABLE 1:** Immunohistochemical findings

Stain	Reaction
PAX8	Negative
AE1/3	Focally weakly positive
EMA	Focally positive
CAM5.2	Focally positive
Chromogranin A	Focally positive (10%–15%)
Synaptophysin	Negative
Vimentin	Negative
CD10	Negative
CD56	Negative
p53	Negative
Bcl‐10	Focally weakly positive
β‐catenin	Positive (membrane)
Ki‐67	90% positive
Trypsin	Positive

Abbreviations: AE1/3, cytokeratin AE1/AE3; Bcl‐10, B‐cell lymphoma/leukemia 10; CAM5.2, cytokeratin CAM5.2; EMA, epithelial membrane antigen; PAX8, paired box gene 8.

**FIGURE 4:**
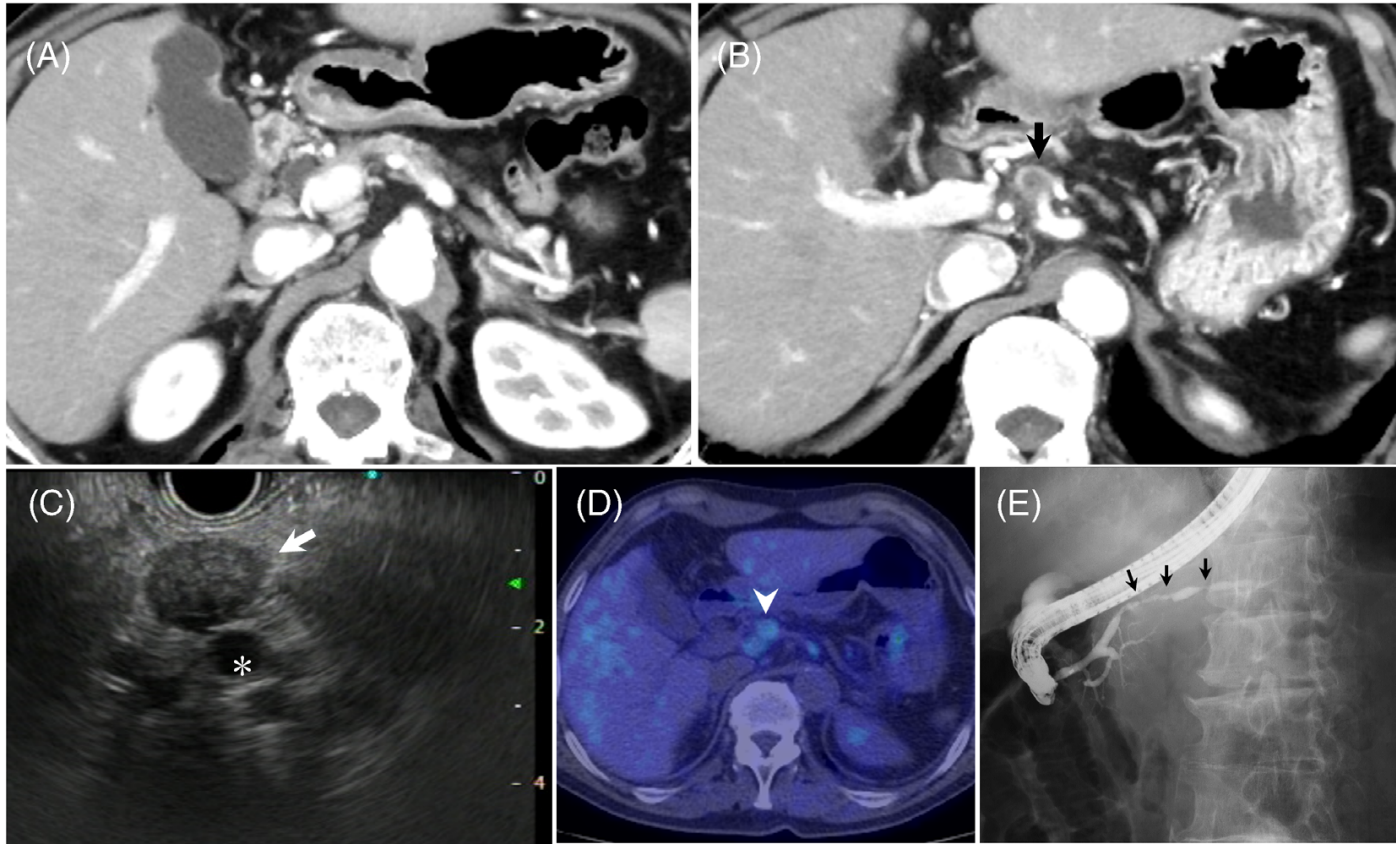
Imaging findings after 18 courses of FOLFIRINOX. (A) Abdominal enhanced computed tomography (CT) showing no tumor in the pancreas. (B) The tumor size is decreased to 15 mm, and it is located at the superior portion of the common hepatic artery (CHA; *arrow*). (C) Endoscopic ultrasonography showing a hypoechoic mass (*arrow*) only around the CHA (*). (D) [^18^F] fluorodeoxyglucose (FDG) positron emission tomography‐CT showing decreased FDG uptake (maximum standardized uptake value: 2.7) in the shrunken tumor (*arrowhead*) (E) Endoscopic retrograde pancreatography showing a difference in the diameter of the main pancreatic duct between the pancreatic body and tail (*arrows*). FOLFIRINOX: 5‐fluorouracil, leucovorin, irinotecan, and oxaliplatin

Thus, we diagnosed the pancreatic tumor as unresectable PACC with a solitary peritoneal dissemination. Thereafter, FOLFIRINOX therapy was initiated. After four courses of FOLFIRINOX, the serum lipase level normalized. After 18 courses, the tumor size was decreased to approximately 15 mm. Of note, no tumor was observed at the pancreatic head (Figure 4A) and the shrunken tumor was observed at the superior portion of the CHA on CT (Figure 4B) and EUS (Figure 4C). FDG‐PET‐CT revealed that the post‐chemotherapy FDG uptake (SUVmax: 2.7) in the shrunken tumor was lower than the pre‐chemotherapy uptake (SUVmax 7.6) and there was no uptake in the pancreas and other organs (Figure 4D).

However, the patient experienced nausea and his tolerance to the treatment decreased. He consented to undergo conversion surgery after being informed of the potential benefits and risks. Preoperative endoscopic retrograde pancreatography revealed a difference in the diameter of the MPD between the pancreatic body and the tail (Figure 4E). Moreover, the pre‐chemotherapy examinations had revealed that the pancreatic tumor had intraductal growth into the pancreatic tail, with FDG uptake observed in the pancreatic tail and the pancreatic tail being hypoplastic at the splenic hilum. Therefore, we decided to perform a total pancreatectomy with regional lymph node dissection 12 months after the initiation of chemotherapy. Intraoperatively, the pancreatic head was easily detached from the PV and SMV, and no vascular resection was necessary. Postoperatively, the patient experienced central venous port infection, but his recovery was otherwise uneventful and he was discharged 31 days postoperatively.

**FIGURE 5:**
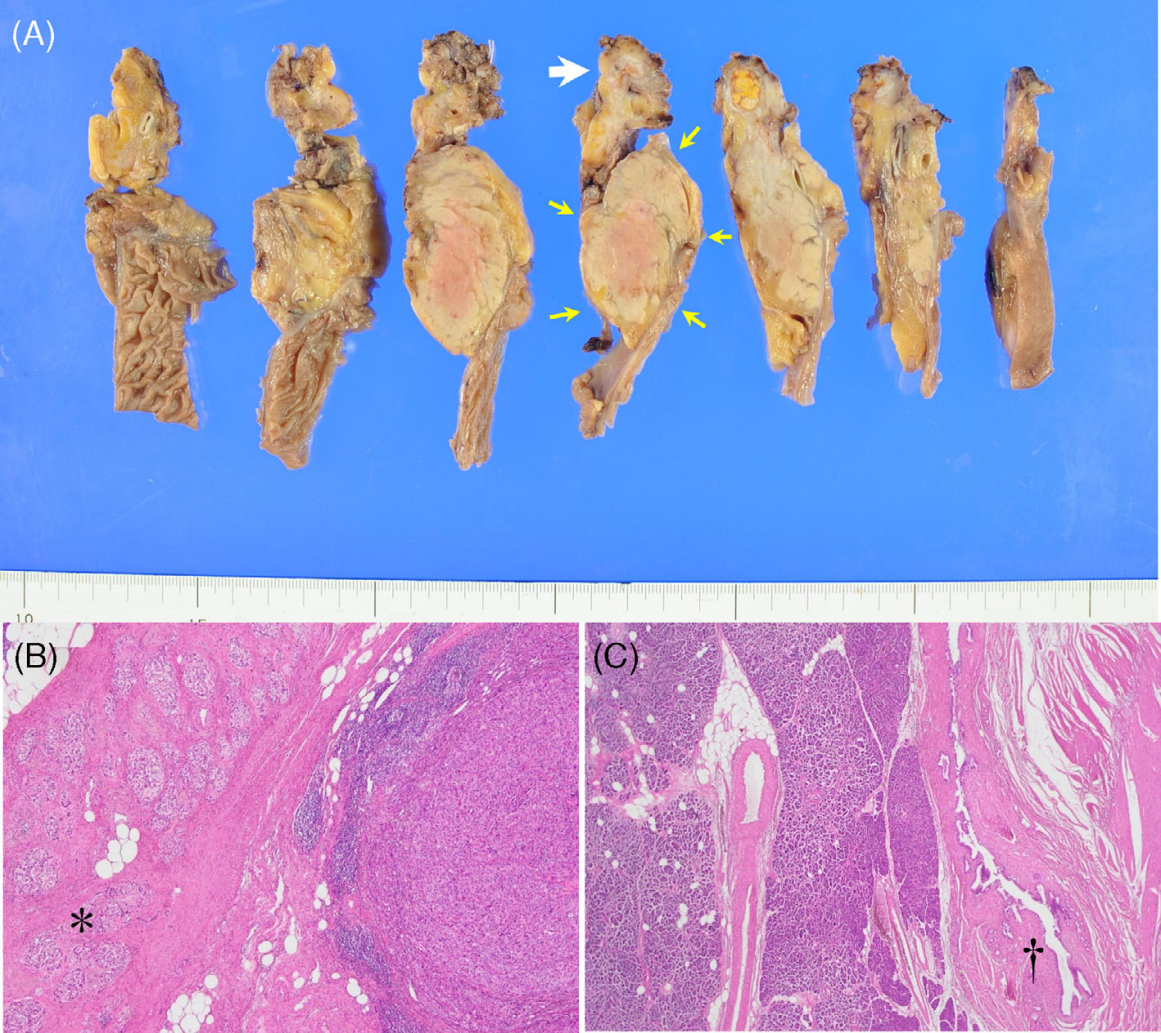
Resected specimen and histopathological examination. (A) Macroscopically, a white nodule (*arrow*) is seen away from the pancreas (*yellow arrows*). (B) Hematoxylin and eosin staining of the white nodule reveals a confluent multinodular lesion consisting of cubic tumor cells with swollen round nuclei. A tendency for glandular formation is seen in some areas, and no lymphovascular or perineural invasion is observed. Although the radial tumor is clearly separated from the pancreas, pancreatic tissue (*) is seen around the residual tumor. (C) No cancer cells are seen within the pancreatic parenchyma and the main pancreatic duct (†)

Macroscopically, the residual tumor was 11 mm in size. It was located away from the pancreas (Figure 5A). The pathological findings confirmed the initial diagnosis of PACC (Figure 5B), and the margin was negative for cancer cells; therefore, conversion surgery was performed on R0 resection. Although the residual tumor was clearly isolated from the main pancreas, pancreatic tissue was observed around the residual tumor, and it was unclear whether the residual tumor was a lymph node because the subcapsular lymphatic vessels were not evident. No cancer cells were found within the pancreatic parenchyma and the MPD (Figure 5C). Histological evaluation of the tumor after chemotherapy based on Evans classification revealed that it was grade IIa.^8^ After conversion surgery, the patient was treated with adjuvant chemotherapy with S‐1. Although the patient required insulin self‐injection because of postoperative pancreatic diabetes (HbA1c: 6%–7%), he did not experience any severe hypoglycemic attack. He has not had any recurrence for 32 months with a good general condition, based on monthly follow‐up with physical examinations and laboratory tests, including tumor markers and the serum lipase level, or a CT follow‐up scan every 6 months.

## 3 DISCUSSION

Herein, we present a rare case of PACC that was resected through conversion surgery after chemotherapy with FOLFIRINOX. To the best of our knowledge, based on a rigorous search of the English literature published between 2009 and 2021 in the PubMed database, this is the fourth report of the use of conversion surgery to treat initially unresectable PACC (Table 2).

**TABLE 2:** Four cases of initially unresectable pancreatic acinar cell carcinoma with chemotherapy and conversion surgery reported in the English literature

Author, year [ref. no.]	Age	Sex	Tumor size (mm)	Reason for being unresectable	Chemotherapy	Treatment period (months)	Effect	Surgical procedure	Adjuvant chemotherapy	Outcome
Yamamoto et al.^9^	71	M	35	Dissemination	S‐1	6	PR	DP	S‐1	At 24 months: alive (without recurrence)
Jimbo et al.^10^	56	M	60	Local advancement (celiac axis involvement)	FOLFIRINOX and FOLFOX	6	PR	Appleby procedure	NA	NA
Kida et al.^11^	59	M	45	Local advancement (portal vein embolism)	S‐1 and GEM	8	PR	DP Portal vein resection	S‐1	At 60 months: alive (without recurrence)
Present case	67	M	67	Dissemination	FOLFILINOX	12	PR	TP	S‐1	At 32 months: alive (without recurrence)

Abbreviations: DP, distal pancreatectomy; GEM, gemcitabine; NA, not available; PR, partial response; TP, total pancreatectomy.

Although PACC accounts only for 1% of all malignant pancreatic exocrine tumors,^1^ approximately 50% of patients with PACC have distant metastases at diagnosis.^1^, ^12^ PACC was previously considered to be aggressive because the recurrence rate after radical resection was as high as 72% and the 5‐year survival rate was <6%.^12^, ^13^ However, Wisnoski et al.^1^ reported that the 5‐year survival rate for resectable PACC was 71.6% and that for unresectable PACC was 22%, revealing that PACC was more indolent than PDAC. The inconsistencies in the reported prognosis among studies may reflect differences in tumor advancement and/or advances in chemotherapy, highlighting the importance of preoperative tumor assessment and individualized treatment approach.

Together with this case, only four cases of conversion therapy have been reported in the literature (Table 2).^9^, ^10^, ^11^ The tumor was unresectable due to local advancement in two cases and peritoneal dissemination in two cases, including this case. Yamamoto et al.^9^ reported a case of unresectable PACC with multiple peritoneal dissemination in which radical resection was performed after primary tumor reduction with S‐1 therapy. Jimbo et al.^10^ reported the successful resection of locally advanced PACC with celiac axis involvement after treatment with FOLFIRINOX followed by FOLFOX (5‐FU, leucovorin, and oxaliplatin). All the patients in the aforementioned four cases were treated with 5‐FU‐containing regimens, and conversion surgery was performed after treatment for more than 6 months. Prognosis was described in three reports, and a long‐term prognosis of more than 24 months was observed in all of them. Three patients, including ours, received adjuvant chemotherapy with S‐1.

Although there is consensus that conversion therapy benefits certain patients with PACC, the regimen to be used remains unclear. Based on clinicians' experience with treating PDAC, S‐1 and gemcitabine,^4^ oxaliplatin‐containing regimens,^5^ and FOLFIRINOX^6^, ^7^ are used to treat PACC in clinical practice. Similar to those identified in patients with colorectal cancer, mutations in the APC/β‐catenin pathway, which lead to cancer cell proliferation, have been identified in patients with PACC.^14^, ^15^ Lowery et al.^15^ suggest that a combination of 5‐FU, irinotecan, and oxaliplatin may be effective for treating PACC. Recent reports of the effectiveness of FOLFIRINOX for treating advanced PACC support this assumption.^6^, ^7^ Furukawa et al.^16^ identified that loss of breast cancer susceptibility gene 2 (*BRCA2*) expression is often observed in patients with PACC. Tumors with *BRCA* mutations are considered to be highly susceptible to platinum chemotherapy.^17^ Our patient showed a good response to FOFILINOX, based on which we assume that he had germline loss‐of‐function mutations in *BRCA2*. Further accumulation of similar cases is necessary to determine the clinical benefits of FOLFIRINOX for treating PACC, including the conversion rate, long‐term survival, and safety.

No specific tumor marker that reflects the therapeutic effect has been found for PACC. CA19‐9 was proposed as a potential marker that reflected tumor response to chemotherapy.^18^ In our patient, the serum lipase level was normalized after four courses of FOLFIRINOX, and since then no increase in the serum lipase level and no recurrence have been observed on laboratory and imaging tests, respectively. Yoshihiro et al.^7^ also reported that the elevated serum lipase level was declined after FOLFIRINOX treatment in their patient with advanced PACC. Therefore, the serum lipase level could be a useful marker of tumor response to treatment, at least in combination with other markers.

In conclusion, we present a case of successful conversion surgery after FOLFIRINOX treatment in a patient with PACC with a solitary peritoneal dissemination. FOLFIRINOX may be effective in patients with PACC, and effective chemotherapy for patients with unresectable PACC may increase the success rate of conversion surgery. Conversion surgery after FOLFIRINOX therapy may be applicable to selective patients, such as in our case of PACC with a solitary peroneal dissemination.

## AUTHOR CONTRIBUTIONS

All authors had full access to the data in the study and take responsibility for the integrity of the data and the accuracy of the data analysis. Conceptualization, S.U., H.M., K.U. and K.H.; Investigation, S.U.; Formal Analysis, S.U.; Resources, H.M., T.K. and M.I.; Data Curation, S.U.; Writing ‐ Original Draft, S.U. and H.M.; Writing ‐ Review & Editing, S.U., H.M., N.T., S.Y., M.M., H.K., T.N., S.Y., T.K., M.I., K.U. and K.H.; Supervision, H.M., T.N., K.U. and K.H.; Project Administration, S.U. and K.H.

## CONFLICT OF INTEREST

The authors have stated explicitly that there are no conflicts of interest in connection with this article.

## ETHICS STATEMENT

All procedures followed have been performed in accordance with the ethical standards laid down in the 1964 Declaration of Helsinki and its later amendments. Written informed consent for publication has been obtained from the patient.

## Data Availability

Data sharing is not applicable to this article as no new data were created or analyzed in this study.
